# Acid base and metabolic parameters of the umbilical cord blood and cerebral oxygenation immediately after birth

**DOI:** 10.3389/fped.2024.1385726

**Published:** 2024-03-28

**Authors:** Martin Dusleag, Berndt Urlesberger, Bernhard Schwaberger, Nariae Baik-Schneditz, Christoph Schlatzer, Christina H. Wolfsberger, Gerhard Pichler

**Affiliations:** ^1^ Division of Neonatology, Department of Paediatrics and Adolescent Medicine, Medical University of Graz, Graz, Austria; ^2^ Research Unit for Neonatal Micro- and Macrocirculation, Department of Paediatrics and Adolescent Medicine, Medical University of Graz, Graz, Austria

**Keywords:** neonates, acid-base and metabolic parameters, cerebral oxygenation, immediate neonatal transition, near-infrared spectroscopy

## Abstract

**Objective:**

Aim was to investigate whether acid-base and metabolic parameters obtained from arterial umbilical cord blood affect cerebral oxygenation after birth in preterm neonates with respiratory support and in term neonates without respiratory support.

**Study design:**

This was a post-hoc analysis of secondary outcome parameters of a prospective observational study including preterm neonates with and term neonates without respiratory support. Non-asphyxiated neonates with cerebral oxygenation measured with near-infrared spectroscopy during the first 15 min and with blood gas analyses from arterial umbilical cord blood were included. Arterial oxygen saturation (SpO_2_) and heart rate (HR) were monitored with pulse oximetry. Potential correlations were investigated between acid-base and metabolic parameters (pH-value, bicarbonate, base-excess, and lactate) and crSO_2_/cFTOE 5 min after birth.

**Results:**

Seventy-seven neonates were included: 14 preterm neonates with respiratory support (mean gestational age [GA] 31.4 ± 4.1 weeks; mean birth weight [BW] 1,690 ± 640 g) and 63 term neonates without respiratory support (GA 38.7 ± 0.8 weeks; BW 3,258 ± 443 g). Mean crSO_2_ 5 min after birth was 44.0% ± 24.2% in preterm and 62.2% ± 20.01% in term neonates. Mean cFTOE 5 min after birth was 0.46 ± 0.06 in preterm and 0.27 ± 0.19 in term neonates. In preterm neonates with respiratory support higher lactate was significantly associated with lower crSO_2_ and SpO_2_ and tended to be associated with higher cFTOE. In term neonates without respiratory support no significant correlations were found.

**Conclusion:**

In non-asphyxiated preterm neonates with respiratory support, lactate levels were negatively associated with crSO_2_ and SpO_2_, whereas in term neonates without respiratory support no associations were observed.

## 1 Introduction

The transition from fetus to newborn represents a highly complex physiological process (1). Initial clinical assessment of the newborn is routinely performed using the Apgar score introduced by Virginia Apgar in 1953 (2). Especially in preterm infants, monitoring by pulse oximeter and/or electrocardiogram is recommended to ensure continuous monitoring of heart rate (HR) and arterial oxygen saturation (SpO_2_) (3–5).

However, brain monitoring is not routinely used yet, even though the brain is the organ, which might be mostly affected by hypoxia. Brain function of the newborn immediately after birth is usually assessed just by clinical evaluation of muscle tone and reflexes (3). Monitoring systems such as Doppler ultrasonography and electroencephalography are of limited value due to their lack of feasibility during immediate neonatal transition (3, 6). Near-infrared spectroscopy (NIRS) represents a method with increasing importance to measure cerebral oxygenation. Based on different absorption spectra of oxygenated and deoxygenated hemoglobin regarding the emitted infrared light, relative changes of these parameters can be measured and cerebral regional oxygen saturation (crSO_2_) can be derived. Further, cerebral fractional tissue oxygen extraction (cFTOE) can be calculated using the equation (SpO_2_-crSO_2_)/SpO_2_ (7). This non-invasive continuous monitoring of crSO_2_ represents a mixed arterial, capillary, and particularly venous saturation and reflects the balance between oxygen supply and oxygen consumption of the brain (6, 8). Within the first minutes after birth, crSO_2_ has been shown to rise to a plateau earlier than peripheral arterial oxygen saturation in term infants, even if the data on this is not fully conclusive (8, 9). In addition, crSO_2_-guided therapy in extremely immature neonates results in a significant reduction in cerebral hyperoxia and hypoxia, although this is not associated with a significantly lower incidence of death and brain injury (8, 10, 11).

crSO_2_ depends on cerebral blood flow (CBF), which is a result of cerebral perfusion pressure (CPP) and cerebral vascular resistance (CVR), on cerebral oxygen consumption (cVO_2_) and on arterial oxygen content (CaO_2_), which mainly depends on arterial oxygen saturation and hemoglobin level (Hb). Several of these parameters are influenced by acid-base balance (pH, HCO_3_, BE and lactate) (12).

Acid-base parameters of arterial umbilical cord blood such as pH, HCO_3_, BE and lactate provide information about the metabolic status of neonates at birth. Severe metabolic acidosis—defined by a pH below 7.0 and a base deficit above 12 mmol/L—is associated with increased neonatal mortality and morbidity (13). Acid-base parameters obtained from venous umbilical cord blood show higher pH values than arterial umbilical cord blood due to the placental origin, whereas samples from heel capillary blood usually indicate lower pH values than arterial umbilical cord blood because of poor peripheral microcirculation (14–16).

The aim of the present study was to analyze the possible impact of acid-base status, measured from a blood sample of the umbilical artery at birth on cerebral oxygenation 5 min after birth in non-asphyxiated preterm neonates with respiratory support and in stable term neonates without respiratory support.

## 2 Material and methods

### 2.1 Design

This was a post-hoc-analysis of secondary outcome parameters of prospective observational studies conducted from October 2015 to September 2018 at the Division of Neonatology, Department of Pediatrics and Adolescent Medicine, Medical University of Graz, Austria. The Regional Ethics Committee approved the study and written parental consent was obtained before birth (EC number: 27–465 ex 14/15).

### 2.2 Inclusion and exclusion criteria

Preterm neonates with respiratory support and stable term neonates without respiratory support delivered by Cesarean section, who were included in the prospective observational study were eligible. Neonates with missing data of the monitoring parameters or acid-base and metabolic parameters were excluded. Neonates with major congenital malformations, and laboratory signs of asphyxia (umbilical artery pH <7.0, BE >12 mml/L or Lactate >2.9 mmol/L) were excluded.

### 2.3 Measurements during immediate transition

After delivery, the neonates were immediately taken to the resuscitation desk and placed supine under an overhead heater. Plastic wraps were used to prevent hypothermia in preterm neonates <29 weeks' gestation. Neonatal stabilization was performed according to neonatal resuscitation guidelines (4, 5) by specialized resuscitation teams (neonatologist/experienced resident and nurse) who were not involved in the study. Respiratory support was provided by continuous positive airway pressure or positive pressure ventilation using a T-piece device (Neopuff Infant Resuscitator, Fisher & Paykel Healthcare, Auckland, New Zealand). Oxygen levels were titrated according to neonatal resuscitation guidelines (4, 5). SpO_2_ and HR were monitored by a pulse oximetry probe (IntelliVue MP30 Monitor, Philips, Amsterdam, The Netherlands) placed around the right wrist/hand. CrSO_2_ was measured using an INVOS 5100 monitor (Covidien, Minnesota, USA) with a neonatal sensor fixed on the left forehead of each neonate. Averaging time of SpO2, HR and crSO2 was 8 s and values were stored every second. cFTOE was calculated with the following formula: (SpO_2_-crSO_2_)/SpO_2_. Cerebral oxygenation was monitored during the first 15 min after birth. Measurements were recorded by the multi-channel system alpha-trace digital MM (BEST Medical Systems, Vienna, Austria) for subsequent analyses. The acid-base and metabolic parameters from arterial umbilical cord blood were analysed with a blood gas analyser (ABL 800 Flex; Fa.Drott, Wiener Neustadt, Austria).

### 2.4 Statistical analysis

Demographic and clinical data are presented as mean ± standard deviation (SD) or median with interquartile range (IQR), as appropriate. Data of preterm neonates with respiratory support and term neonates without respiratory support were compared using t-test for independent samples, Mann-Whitney-U or Chi-square test as appropriate.

Mean of values of minute 5 after birth of SpO_2,_ HR, crSO_2_ and cFTOE values were correlated to pH, bicarbonate (HCO_3_), base excess (BE) and lactate using Spearman's rank correlation coefficient or Pearson's correlation, as appropriate. This time-point was chosen to be close to the blood sample time-point. The analyses were performed in an explorative sense. Therefore, no correction for multiple testing was performed.

A *p*-value <0.05 was considered statistically significant. The statistical analyses were performed using IBM SPSS Statistics 27.0.0 (IBM Corporation, Armonk, NY, USA).

## 3 Results

**Figure 1:**
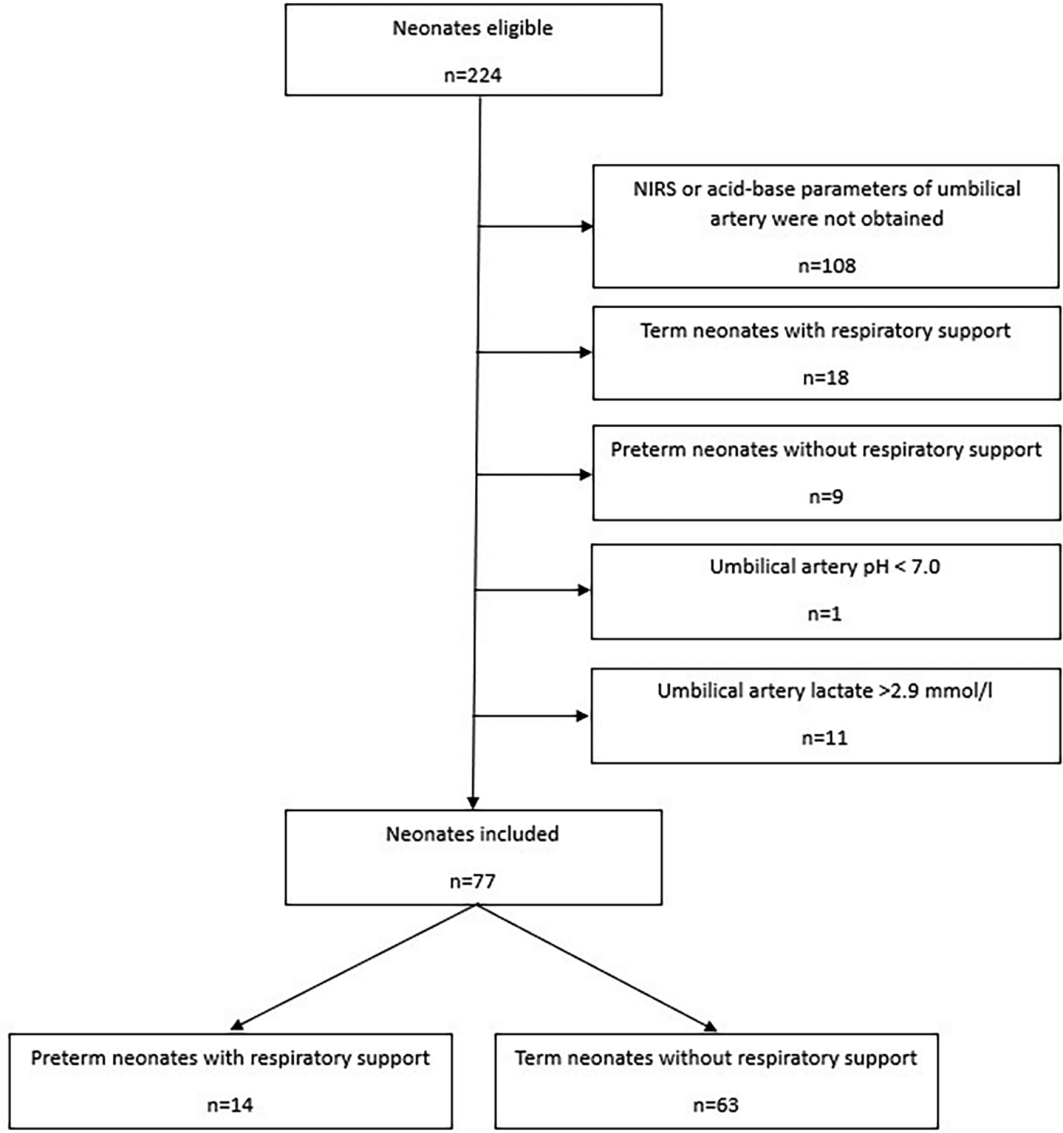
Patient flow diagram. NIRS, near infrared spectrometry.

Out of 224 eligible neonates 77 were included (Figure 1): 14 preterm neonates with respiratory support and 63 stable term neonates without respiratory support. Most neonates were excluded due to missing NIRS data at minute five or missing acid-base and metabolic parameters of umbilical artery. Demographic data of preterm and term neonates are presented in Table 1.

**Table 1:** Demographics, monitoring data 5 min after birth and acid base metabolism of the umbilical artery in preterm neonates with respiratory support and stable term neonates without respiratory support.

		Preterm (*n* = 14)	Term (*n* = 63)	*p*-value
Demographics	Gestational age (weeks)	31.4 ± 4.1	38.7 ± 0.8	<0.001
	Birth weight (g)	1.690 ± 640	3.258 ± 443	<0.001
	Female sex (%)	4 (29)	24 (38)	0.693
	Apgar 1 min	8 (6.75–8)	9 (9–9)	<0.001
	Apgar 5 min	9 (8–9)	10 (10–10)	<0.001
	Apgar 10 min	9 (9–9.25)	10 (10–10)	<0.001
Monitoring	SpO_2_ (%)	66 ± 12	83 ± 9	<0.001
	Pulse (bpm)	148 ± 16	154 ± 17	0.275
	CrSO_2_ (%)	44.0 ± 24.2	62.2 ± 20.01	0.012
	FTOE	0.46 ± 0.2	0.27 ± 0.19	0.011
	pH	7.32 ± 0.06	7.32 ± 0.03	0.815
Acid-base metabolism	Bicarbonate (mmol/L)	22.9 ± 2.5	23.0 ± 1.20	0.839
	Base excess (mmol/L)	0.70 ± 2.89	1.37 ± 1.55	0.418
	Lactate (mmol/L)	2.6 ± 1.5	1.7 ± 0.42	0.012

Data are presented as mean values ± SD or median (IQR). SpO_2_, arterial oxygen saturation; CrSO_2_, cerebral regional oxygen saturation; FTOE, fractional tissue oxygen extraction.

Concerning monitoring parameters (Table 1) preterm neonates receiving respiratory support showed significantly lower values of crSO_2_ and SpO_2_ and higher values in cFTOE than term neonates without respiratory support, whereas there was no statistically significant difference in HR. Besides lactate levels, which were statistically significantly higher in preterm neonates with respiratory support, no statistically significant differences were observed regarding pH, HCO_3_ or BE between the two groups (Table 1).

In preterm neonates with respiratory support lactate levels correlated significantly negatively with crSO_2_ and SpO_2_ 5 min after birth (Table 2). In term neonates no correlations were observed.

**Table 2:** Correlation analyses of SpO_2_, pulse, CrSO_2_ and FTOE measured 5 min after birth with umbilical artery pH, bicorbonate, base excess and lactate and in preterm neonates with respiratory support and stable term neonates without respiratory support.

		pH		Bicarbonate		Base excess		Lactate	
		*r*	*p*-value	*r*	*p*-value	*r*	*p*-value	*r*	*p*-value
Preterm (*n* = 14)	SpO_2_	0.402	0.154	0.051	0.863	0.013	0.964	−.540*	0.046
	HR	−0.167	0.644	−0.248	0.489	−0.367	0.267	0.146	0.687
	crSO_2_	0.461	0.131	0.344	0.273	0.253	0.428	−0.589*	0.044
	FTOE	−0.38	0.279	0.003	0.994	−0.039	0.914	0.59	0.073
Term (*n* = 63)	SpO_2_	−0.008	0.952	0.104	0.423	0.145	0.261	0.112	0.393
	HR	−0.118	0.368	−0.112	0.398	−0.149	0.256	0.257	0.052
	crSO_2_	0.085	0.515	0.139	0.290	0.085	0.514	0.024	0.854
	FTOE	−0.19	0.147	−0.133	0.316	−0.068	0.603	0.077	0.566

SpO_2_, arterial oxygen saturation; CrSO_2_, cerebral regional oxygen saturation; FTOE, fractional tissue oxygen extraction.

*p-value <0.05.

## 4 Discussion and conclusion

In the present study we have demonstrated that crSO_2_ and SpO_2_ 5 min after birth were associated with lactate levels in compromised preterm neonates with respiratory support, whereas in stable term neonates without respiratory support no associations were observed. In addition, lactate in preterm neonates also tended to be associated with cFTOE. Apart of lactate no further parameters of acid-base status were associated with SpO2, HR, crSO2 or cFTOE, neither in preterm neonates with respiratory support nor in stable term neonates.

SpO_2_ represents the percentage of hemoglobin saturated with oxygen in peripheral arterial blood (17). Our findings regarding SpO_2_ values in term neonates 5 min after birth are consistent with those of Rabi et al. (18), who described median SpO_2_ values of 81% (75–83).

Lactate is produced as a metabolite in anaerobic glycolysis and is elevated in case of impaired cellular oxygenation (19). The correlations in the present study were observed despite the fairly narrow lactate range within the study population with values <2.9 mmol/L. Similar findings with negative correlations between lactate levels obtained from capillary blood and crSO_2_ and with positive correlations between lactate levels and cFTOE in preterm neonates 15 min after birth were published by Mattersberger et al. (20) as well as by Janaillac et al. (21) in extremely preterm infants in the first 72 h after birth. The observed correlations could be explained by the fact that lactic acidemia induces pulmonary vasoconstriction and decreases cardiac stroke volume as a result of impaired cardiac contractility (22). Subsequently, low cardiac output leads to reduced oxygen supply of peripheral tissue and diminished peripheral oxygen saturation. The association between elevated lactate levels and poor hemodynamics has also been described in previous studies (23, 24). Considering lactate as a parameter of hemodynamics, the observed association between lactate levels and cerebral oxygenation may reflect impaired cerebrovascular autoregulation in preterm neonates. Intact cerebrovascular autoregulation allows constant CBF over a wide range of blood pressure, resulting in CBF independency of CPP. Impaired cerebrovascular autoregulation leads to a pressure-dependent CBF due to its linear correlation with CPP, resulting in deleterious CBF fluctuations in response to CPP variations (12, 25–28). Several studies have already indicated impaired cerebrovascular autoregulation in compromised preterm neonates (25–27, 29–33). Considering that in the present study cFTOE also tends to be associated with lactate in preterm neonates, low crSO_2_ may not only be a result of diminished oxygen content -in terms of low SpO_2_-, but also due to increased oxygen extraction in the cerebral microcirculation. In contrast, studies of infants diagnosed prenatally with congenital heart disease (34) and of critically ill neonates and of infants without cerebral damage (35) do not indicate associations between lactate levels and cerebral oxygenation. However, they showed negative correlations between cerebral oxygenation and SpO_2_. Since these studies included cerebral oxygenation measurements beyond the immediate fetal to neonatal transition and differentiation between preterm and term neonates was not considered, the differences in their findings could be result of a better cerebral autoregulation.

As far as other acid-base and metabolic parameters are concerned, these were slightly more alkaline compared to previous publications, which mostly included neonates born by vaginal delivery (19, 36). This is most probably due to the effect that neonates with laboratory signs of asphyxia were excluded, since we wanted to analyse the effect of blood gases and lactate in non-asphyxiated neonates. Furthermore, differences might be due to differences in the metabolic stress of the fetus caused by repeated uterine contractions during vaginal delivery (19) and Cesarean section.

Measurement of umbilical cord blood pH, HCO_3_ and BE provides essential information about acid-base metabolism and extrauterine adaption of newborns (13). In accordance to the present study no correlations between crSO_2_ and pH were found in children during cardiopulmonary bypass surgery (37), in infants diagnosed prenatally with congenital heart disease during the first 72 h (34) and in stable preterm neonates in neonatal intensive care (38). HCO_3_ is the physiologically most important buffer system in the human body (39). BE is used for quantification of changes in metabolic acid-base status and together with umbilical artery pH it is crucial to estimate the risk for newborn cerebral damage (40, 41). Mattersberger et al. (20) indicated positive correlations between HCO_3_ measured 15 min postnatally and cFTOE in term neonates and correlations of lower pH and BE also measured 15 min postnatally with lower crSO_2_ and higher cFTOE in preterm neonates. A point of innovation of the present study compared to Mattersberger et al. is the fact that by using umbilical cord blood the blood sampling is immediately after birth and might help to predict changes in the transition period and especially predict cerebral oxygenation in this vulnerable period (42). Furthermore capillary blood gas measurements do not always predict arterial blood gas values in an accurate way (13). In contrast, neither in stable preterm neonates in neonatal intensive care (38) nor in critically ill neonates and infants without cerebral damage (35) correlations were found between crSO_2_ and BE. Aldrich et al. (43) showed, that crSO_2_ is positively correlated with pH and negatively with BE shortly before birth and negative correlations between crSO_2_ and BE and HCO_3_, respectively were reported by Nissen et al. (44) in term-born infants with hypertrophic pyloric stenosis. A possible explanation of the differences to our findings are different study populations, comorbidities but also the fact, that in contrast to the present study, blood gas analysis were obtained from capillary blood. Samples obtained from arterial blood or umbilical cord blood tend to have more alkaline pH values than those of capillary blood (45). The present study was a post-hoc-analysis with all its inherent limitations. The small sample size of preterm neonates with respiratory support and possible interactions between the investigated acid-base and metabolic parameters and cerebral oxygenation mark further shortcomings. However, the described correlations in preterm neonates could be interpreted as an impaired cerebral autoregulation and are therefore an important finding.

In conclusion, in non-asphyxiated preterm neonates with respiratory support lactate levels were significantly associated with crSO_2_ and SpO_2_, whereas in stable term neonates without respiratory support no associations were observed.

Future studies will be necessary to evaluate causal relationships between acid-base and metabolic parameters and cerebral oxygenation during the period of transition from fetal to neonatal life.

## Data Availability

The raw data supporting the conclusions of this article will be made available by the authors, without undue reservation.
